# A proteomic study of cMyc improvement of CHO culture

**DOI:** 10.1186/1472-6750-10-25

**Published:** 2010-03-22

**Authors:** Darrin Kuystermans, Michael J Dunn, Mohamed Al-Rubeai

**Affiliations:** 1 School of Chemical and Bioprocess Engineering, University College Dublin, Belfield, Dublin 4, Ireland; 2 UCD Conway Institute of Biomolecular and Biomedical Research, University College Dublin, Belfield, Dublin 4, Ireland

## Abstract

**Background:**

The biopharmaceutical industry requires cell lines to have an optimal proliferation rate and a high integral viable cell number resulting in a maximum volumetric recombinant protein product titre. Nutrient feeding has been shown to boost cell number and productivity in fed-batch culture, but cell line engineering is another route one may take to increase these parameters in the bioreactor. The use of CHO-K1 cells with a *c-myc *plasmid allowing for over-expressing c-Myc (designated cMycCHO) gives a higher integral viable cell number. In this study the differential protein expression in cMycCHO is investigated using two-dimensional gel electrophoresis (2-DE) followed by image analysis to determine the extent of the effect c-Myc has on the cell and the proteins involved to give the new phenotype.

**Results:**

Over 100 proteins that were differentially expressed in cMycCHO cells were detected with high statistical confidence, of which 41 were subsequently identified by tandem mass spectrometry (LC-MS/MS). Further analysis revealed proteins involved in a variety of pathways. Some examples of changes in protein expression include: an increase in nucleolin, involved in proliferation and known to aid in stabilising anti-apoptotic protein mRNA levels, the cytoskeleton and mitochondrial morphology (vimentin), protein biosysnthesis (eIF6) and energy metabolism (ATP synthetase), and a decreased regulation of all proteins, indentified, involved in matrix and cell to cell adhesion.

**Conclusion:**

These results indicate several proteins involved in proliferation and adhesion that could be useful for future approaches to improve proliferation and decrease adhesion of CHO cell lines which are difficult to adapt to suspension culture.

## Background

Chinese hamster ovary cells (CHO) are the most popular commercial platform for the production of therapeutic proteins with much development going into the use of such cell lines for increasing product yields. Hence the productivity of cell cultures has improved more than 100-fold in the last two decades mainly due to developments of fed-batch culture systems, media and process optimisations in conjunction with expression technologies [1,2]. As much as improvements in specific productivity (Q_p_) are important in cell lines, growth characteristics also have a significant impact on the process. A good cell line proliferative capacity and a high integral viable cell number (IVC) can result in high volumetric recombinant protein production rates. Thus the mammalian biopharmaceutical industry has research interests directed towards the development of cell lines with high proliferation rate that can be grown to high densities and have high production capabilities. Induction of the transcription factor Myc promotes cell proliferation and transformation by activating growth promoting genes or by repressing the expression of growth arrest genes [3-11]. The gene, *c-myc*, is a prime candidate that regulates cell proliferation in such a way that its introduction into cell lines may be advantageous.

Research has shown that transfection of adherent CHO cell line with the *c-myc *gene resulted in increased proliferation rate and cell number [11,12]. To understand the cellular activity that results from the overexpression of c-Myc (via the transfection with c-myc plasmid) in CHO cells, the techniques of two-dimensional polyacrylamide gel electrophoresis (2-DE) and statistically viable image analysis combined with mass spectrometry were employed to help identify the proteins involved. This technique allows for the separation of complex protein mixtures with a relative high resolution involving a two-step separation of the proteins, first by isoelectric point and then by size to generate protein maps of the investigated proteome.

Currently, the database of the proteomes of CHO cell is not complete, but due to similarities of mammalian proteins between species successful identification of proteins can be done across species [13]. This has made it possible to carry out several proteomic studies on the CHO cells including a general proteome map [14,15]. Further analysis of the protein regulation under controlled conditions has led to the 2D proteome analysis of CHO cells in response to hyperosmotic conditions [16], increased production levels[17,18], low temperature shift [19], and growth factor stimulation [20]. Also the proteomic work carried out on c-Myc is limited, not in CHO cells, and none has been done using the 2DE approach[21,22] In this study, it is the objective to identify potential proteins involved in producing the phenotype seen with a c-myc plasmid in CHO cells in the hope of increasing our understanding of the intracellular and physiological changes and providing further insights into possible CHO cell manipulation for improved cell line development. In this work the cell line containing c-myc plasmid is compared to the parental cell line to determine if any bioprocessing benefit would have been achieved. It may be useful to state that a comparison of the cells containing the *c-myc *plasmid with cells containing blank plasmid would provide further specific information on the c-myc effects.

## Methods

### Cell Transfection and Maintenance

The transfection and selection protocol has been previously reported [12]. Briefly, the cMycCHO (*c-myc *plasmid containing CHO-K1) cell line resulted by calcium-mediated stable transfection of CHO-K1, with the DORclaG123 (c-Myc plasmid). The plasmid was kindly donated by Dr. T. Littlewood (then at Imperial Cancer Research Fund, UK). Surviving cells were pooled separately from each transfection and maintained in Ham's F12 supplemented with 5% FCS. Dilution cloning was used to select a stable cMycCHO clone with high over expression of the *c-myc *gene for subsequent experiments. Selection pressure was maintained by incubation in DMEM/F12 with 5% FBS (Lonza Biologics) and 1 mg/mL geneticin G418 (Sigma Aldrich, UK) every 10^th ^generation. Samples were taken before and after incubation to ensure stable overexpression of the c-Myc protein. To negate the effects of the antibiotic selection pressure was removed 2 passages before an experimental run.

### Static Batch Culture of CHO-K1 and cMycCHO

In subsequent experiments CHO-K1 and cMycCHO were maintained in DMEM/F12 with 5% FBS (Lonza Biologics, UK) at 37°C vented in 5% CO_2_ incubator. Static cultures from each cell line were plated in 25 cm^2 ^(75 cm^2 ^for proteome isolation) vented T-flasks with an initial density of 2 × 10^5 ^cells/ml allowing triplicate flasks to be sacrificed at regular intervals for each cell line. Viable cell concentration and viability were monitored using the trypan blue exclusion method [23]. The sample for proteomic analysis was taken at late exponential phase of the culture. This point also put emphasis on that in a limited nutrient environment the cMycCHO can maintain a higher cell density. To verify the inoculum had c-Myc over expression a separate duplicate culture was run for Western analysis.

### Determination of Extracellular Glucose and Lactate

Glucose was measured with an Ascencia contour (Bayer Diagnostics, Ireland) glucose meter using pre-calibrated Mircofill test strips (Bayer Diagnostics, Ireland). Lactate was measured using the Accutrend Lactate Meter together with BM-Lactate Strips (Roche Diagnostics GmbH, Germany). Briefly, The BM-Lactate calibration strip was used to calibrate the instrument to the accompanied BM-Lactate strips. Lactate was determined by reflectance photometry at a wave length of 657 nm via a colorimetric-oxidase mediator reaction.

### Proteome Isolation

**Figure 1:**
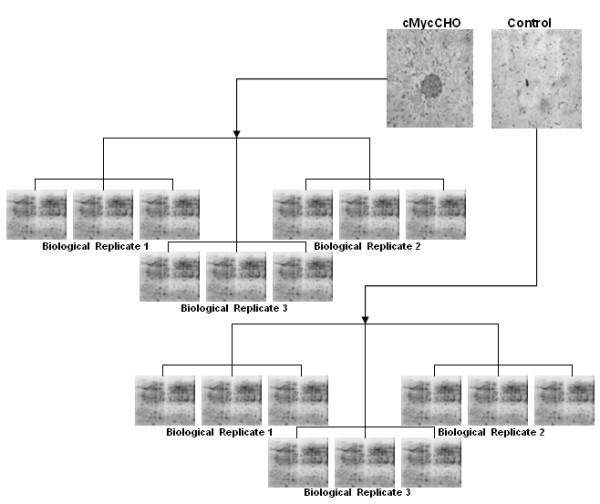
**Experimental setup and inverted microscopic image of adherent culture of CHO-K1 and cMycCHO cell lines**. The Morphology of cell lines shows adherent c-Myc over-expression causing morphological changes to the cell with the formation of foci. The experimental design above demonstrates that from the 3 biological replica culture samples, 3 technical sample replicates from each of these were used in proteomic image analysis setup.

The experimental setup is illustrated in Figure 1. A minimum of 1 × 10^7 ^cells of each replicate was used to obtain total cellular protein which was washed in isotonic (0.35 M) sucrose to reduce salt contamination and then resuspended in 400 μl of lysis buffer (Ultrapure reagents from GE Healthcare, Uppsala, Sweden): 2% (w/v) CHAPS, 9.5 M urea, 0.8% (w/v) Pharmalyte pH 3-10, 1% (w/v) DTT (GE Healthcare) and 1 protease inhibitor tablet per 10 ml (Roche Molecular, Mannheim, Germany) for 30 minutes. The cells where then centrifuged at 4°C and 17,000 g for 30 minutes. Samples were stored at -80°C for minimum of 24 hours before concentration determination using a modified Bradford assay [24].

### Two-Dimensional Sodium Polyacrylamide Gel Electrophoresis (2-DE)

An amount of 100 ug protein/gel was used for analytical gels and 400 ug protein/gel for preparative gels. Lysed samples were diluted in rehydration buffer (8 M urea, 0.5% [w/v] CHAPS, 0.2% [w/v] DTT, 0.2% [w/v] Pharmalyte pH 3-10); and the appropriate protein load applied to each 24 cm, linear pH 4-7, immobilized pH gradient (IPG) strip (GE Healthcare, Uppsala, Sweden) by in-gel rehydration [25-27]. IPG strips were focused at 50 uA/strip at 20°C for 4 voltage changes, these were; (1) 75,000 Volt-hours (Vh) at 3500 V, followed by (2) a gradient increase to 8000 V for 10 min, which was then left at (3) 8000 V for 1 hour, followed by, (4) 100 V for a maximum of 3 hours using an IPGphor System (GE Heathcare). Subsequently, the IPG strips were once equilibrated in a buffer containing 6 M Urea, 50 mM TrisCl pH 8.8, 30% (v/v) glycerol, 2% (w/v) SDS and 1% (w/v) DTT for 15 min and then in the same buffer but with the addition of 4.8% (w/v) iodacetamide for a further 15 min. Proteins were separated in the second dimension by SDS-PAGE (gel format 24 × 20 × 0.15 cm). After gel fixation in methanol/acetic acid solution, the analytical gels were stained with 2D-Silver Stain II Kit (Daiichi Pure Chemicals, Tokyo, Japan) while the preparative gels were stained with the PlusOne™ Silver Staining Kit (GE Healthcare) using a modified, mass spectrometry compatible protocol [28].

### Image and Data Analysis

2-D gel images were captured using a Molecular Imager GS-800 scanner (Bio-Rad), warped with TT900 S2S software (Nonlinear Dynamics, Newcastle, UK) and quantitatively analysed using Progenesis PG240 SameSpot™ software (Nonlinear Dynamics, Newcastle upon Tyne, UK). Protein spot detection and normalization (an integration of the area and optical density, as a percentage of the total volume of all detected spots) was performed automatically, followed by manual checking of detected spots; reassigning a numerical spot designation and removing background noise before renormalization and applying the SameSpot™ analysis algorithm (SSAA). This procedure, addresses measurement variations from gel to gel while pixel level alignment corrects for positional variation from gel to gel. The fold change value is calculated from the mean normalised volumes between cMycCHO and CHO-K1. All spots were compared to control reference gel and for eliminating experimental discrepancies between spots. SSAA applies the same spot outline to corresponding spots for all gels before the application of ANOVA (p < 0.05) for statistically significant changes in their protein expression based on the normalized volume of each spot. Further ontology analysis was carried out by transferring the Progenesis data to GenePilot™ V1.95b (TG Services, Inc. CA USA).

### LC-MS/MS and Data Analysis

Manual excision of the protein spots of interest and tryptic digestion were carried out using an Ettan Digester (GE Healthcare, Uppsala, Sweden) and peptide analysis was carried out using a linear ion trap mass spectrometer (LTQ Mass Spectrometer; Thermo-Finnigan Ltd, Hemel Hempstead, UK). The mass spectra parameters were set to a full scan MS mass range of 400-1600 m/z. The mass spectrometer was equipped with a nanospray ion source (Thermo Electron Corporation, Waltham, MA) with a spray voltage of 1.9 kV. BioWorks 3.2 software (Thermo Electron Corporation), featuring the TurboSEQUEST search algorithm, was used to query the Swiss-Prot database for protein identification. Two filters were used to consider significant hits, peptide Xcorr (statistical criteria correlation value) versus charge state (charge state: +1, +2, +3; set-points: 1.9, 2.0, 2.5) with a peptide probability-based scoring algorithm cut-off of < 10^-3^.

### Western Blot

A protein load of 50 μg/well underwent electrophoresis on a 12% sodium dodecyl sulfate-polyacrylamide (SDS-PAGE) mini gel (Pierce, Rockford, IL) subsequent to dilution in 1× Laemli buffer with prior denaturation at 95°C for 10 minutes. The gel was stained with Instantblue™ (Novexin Ltd, UK) for visual verification of loading before de-staining for electrotransfer. The protein was then transferred to an Immobilon™-P membrane (0.45 μm) (Millipore, Cork, Ireland) followed by blocking of the membranes with 5% Marvel-PBS for 1 hour at room temperature and probed overnight at 4°C by incubation with the primary antibody (Santa Cruz Biotechnology, CA), either anti-myc 9E10, rabbit anti-RKIP, rabbit anti-NUCL, rabbit anti-eIF6, or rabbit anti-GRP78. The blot was washed and then incubated with a HRP conjugated anti-mouse (only for anti-myc) or anti-rabbit whole IgG as the secondary antibody. Bands were developed by chemiluminescent detection (SuperSignal West Pico System, Pierce) according to the manufacturer's instructions. Any required blot stripping was carried out by incubating in NaOH followed by an overnight blocking in 10% Marvel-PBS at 4°C. The membranes were stained with Coomassie Brilliant Blue R-250 (Sigma Aldrich, UK) to re-verify loading and uniformity of the electrotransfer.

## Results and Discussion

### Growth of Cell Lines

**Figure 2:**
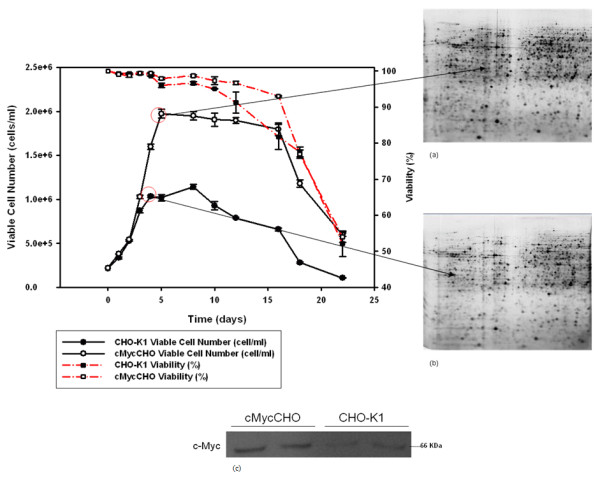
**Growth Curve and 2D gel sample points**. The growth curve of cMycCHO (clear circles) compared to CHO-K1 (Black circles). This culture seeded and grown (n = 3) in parallel, cMycCHO (maximum cell density 1.9 × 10^6 ^cells/ml) has an increased proliferation capacity compared to the CHO-K1 (maximum cell density 1.1 × 10^6 ^cells/ml). The proteomic sampling was done at the late exponential phase to analyse the different proteins involved in developing this phenotype to produce (a) the representative proteome map of cMycCHO from 2D SDS-PAGE image scan and (b) the representative proteome map of CHO-K1 from 2D SDS-PAGE image scan from loading 100 μg of protein per gel. In visual comparisons, it can be recognised that the cMycCHO proteome map shows an overall increase in up-regulated protein expression due to cMyc activity at the late exponential phase of culture. The western (c) indicates a duplicate culture run with the inoculum to verify c-Myc over-expression.

For proteomic profiling, a batch culture, in triplicate was grown (Figure 2) with adherent cMycCHO alongside its control CHO-K1. The cMycCHO culture had an increase in overall proliferative capacity being able to proliferate to higher cell densities of 1.94 × 10^6 ^cells/ml compared to 1.14 × 10^6 ^cells/ml of CHO-K1. The viability remained above 90% for both cell lines until day 10 after which CHO-K1 started a steady decline in viability while cMycCHO culture continued to survive at an average of 93% on day 16 compared to CHO-K1 which had a viability of 82%. Under these growth conditions cMycCHO had an increased growth rate of 0.54 day^-1 ^compared to 0.48 day^-1 ^for CHO-K1. The increase in maximal cell density combined with the increased growth rate, for an 8 day culture period, resulted in an IVC increase of 59% for the cMycCHO cell line reaching 10.54 × 10^9 ^cell day L^-1^. Proteomic samples were taken from where the biggest variation in cell number was seen between the CHO-K1 and cMycCHO while still at the highest viability for both cultures and this was seen to be the late exponential phase.

**Figure 3:**
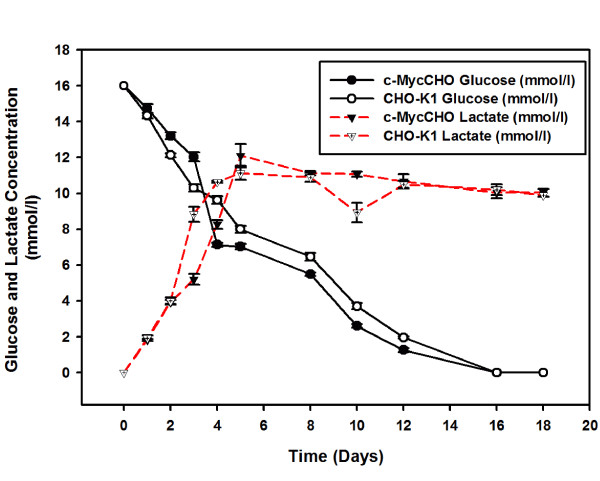
**Glucose utilization and lactate production**. For the cMycCHO culture, glucose utilisation is represented by a solid line with a black circle and a solid line with clear circle for CHO-K1. For lactate measurements, taken from the extracellular environment, the cMycCHO culture is represented by a dashed line with black upside down triangles and CHO-K1 is indicated with a dashed line with clear upside down triangles. Both cultures show a similar profile but differ in specific utilisation and production.

The increased growth rate had an effect on specific glucose utilisation and lactate production (Figure 3). In Figure 3 it can be observed that the glucose and lactate consumption and production profiles of cMycCHO are only slightly greater than those of the control. Thus when calculating these as rates per cell there was a decrease in the specific glucose rate of 1.72 μmole 10^-6 ^cells day^-1^, for cMycCHO, compared to a rate of 2.15 μmole 10^-6 ^cells day^-1 ^for CHO-K1. This shows that the cMycCHO cell line could be considered more efficient at glucose utilisation. Similarly, specific Lactate production by cMycCHO had decreased by 49.5% in comparison to the CHO-K1 value of 4.32 μmole 10^-6 ^cells day^-1^. These results are in agreement with earlier batch culture experiments where both glucose utilisation and lactate production have decreased in the c-myc engineered cell line [12].

### Proteome Profile

**Figure 4:**
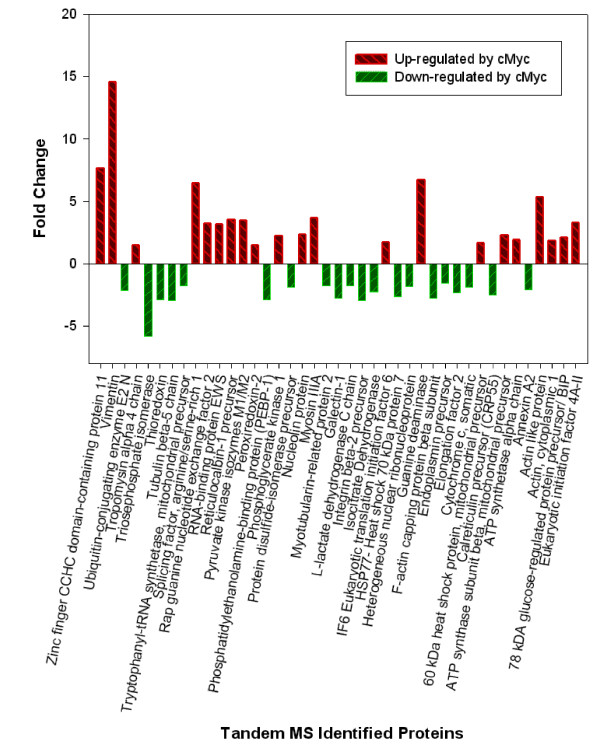
**Barchart of MS/MS identified proteins illustrating preliminary identification of proteins and their corresponding fold changes with a p-value above 0.05**.

**Figure 5:**
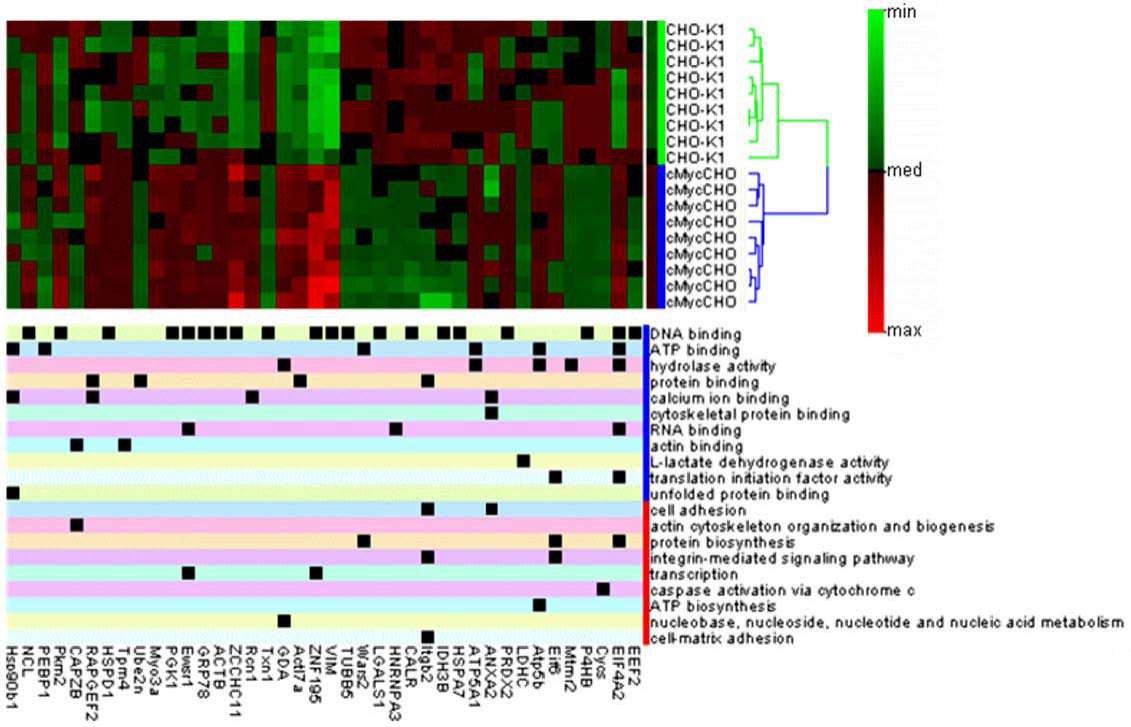
**The Heatmap illustrates the hierarchical clustering found in the replicate profile differentiating the two cell lines for the set of identified proteins found in the protein expression and the associated ontology of the proteins**. The value range is defined by the minimum and maximum values in the entire dataset with zero being the medium value. The row dendigram displays the cluster of the proteome replicates with similar rows clustered together. Distances can be roughly determined by the height of the node that binds the replicates together, all technical replicates were found to be alongside each other making the start of each biological replicate 1^st^, 4^th^, 7^th ^in line. One replicate in the CHO-K1 dataset showed the most variation from the dataset but was still within the CHO-K1 node. The ontology indicated that the c-Myc had affected a large range of molecular pathways and from the identified dataset blue indicates cellular processes affected and red indicates molecular function affected.

The effect of constitutive expression of *c-myc *plasmid on the CHO proteome was fairly extensive with initially more then a 300 proteins deregulated by visual analysis. Using the Progensis™ statistical package, there were 122 spots detected as being differentially expressed from the 18 2-D gel images at high confidence (>95%) for further processing with the tandem MS identification procedure. From the excised spots, 41 came through the proteomics procedural pipeline passing the identification scoring criteria (Additional file 1; Table S1). Figure 4 shows the fold change in regulation for these proteins obtained from the use of Progensis™ image analysis software. The effect of c-Myc on cellular activity is known to be extensive [7] and thus it is important to try to identify, from the list of protein hits, which of these proteins may help confer advantageous phenotypes for cell culture processes, by further investigating the molecular functions. From the protein regulation heat-map, the ontology using the gene information gives both molecular and biological function (Figure 5) which provides the initial indication of the varied functions of the proteins that are deregulated. To do a fully comprehensive analysis of the protein pathway system as a whole, the number of proteins identified would need to be increased. This is due to a bias introduced towards the identification of more highly expressed proteins which are seen using 2D analysis. However, the majority of proteins identified do give a varied ontology makeup thus they may be used as an outline for the pathways affected.

From the proteomic data, the majority of proteins identified can be classified into subcategories of proliferation, protein biosynthesis, energy metabolism, cellular adhesion, protein folding and cytoskeleton although it must be noted that some proteins have multi-functionality and will cross over into other cell signaling pathways such as Nucleolin (NUCL), 60 kDa heat shock protein (HSPD1), and Peroxiredoxin 2 (PRDX2), which all have an additional anti-apoptotic activity. Other proteins identified may not have a specific classified function(s) at this time.

### Proliferation

**Figure 6:**
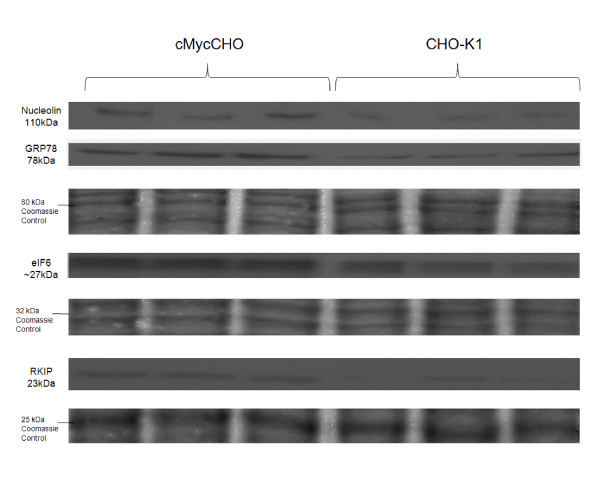
**Immunoblot validation of identified protein spots**. A western analysis of biological triplicate samples with c-Myc over-expression observed in the cMycCHO cell lines validated the observed proteomic results. The membranes were stained with Coomassie brilliant blue to re-verify loading and uniformity of the electro-transfer after band detection, due to c-myc de-regulating both cytoskeleton and metabolic proteins.

The protein, PEBP-1(Phosphatidylethanolamine Binding Protein), also known as Raf kinase inhibitor protein (RKIP) is down regulated in the proteome profile, confirmed by the western blot (Figure 6). It has been implicated as a direct negative regulator of key signaling kinases [29] but seems to function rather as a modulator of the Raf/MEK/ERK kinase signaling cascade rather than an off-switch while its ability to bind nucleotides and opiods mean it may also have other functions [30]. One important fact remains, that its down regulation has implications on mitosis where RKIP depletion and as a result, Raf-1 up-regulation, causes cells to mover faster through mitosis. Its down-regulation has further implications on cancer progression and survival [31,32]. In the case of cMycCHO, RKIP depletion, may help increase the proliferation potential of the cell without the use of growth factors since the Raf/MEK/ERK cascade is known to be stimulated by growth factors [33]. Recently it was shown that ovarian cancer cells undergo growth factor stimulated growth by Raf-1 [34] which is regulated by RKIP [35].

One identified protein of interest is nucleolin (NUCL), which, like c-myc, is a multifunctional protein with increased expression in exponentially growing cells and is found mainly in the nucleolus, at the dense fibrillar granular regions [36,37]. NUCL has recently been discovered to be a direct target of c-myc [22,38], confirming the proteomic results for this protein both in the 2D analysis and the western blot analysis (Figure 6). NUCL is known to be involved in ribosome biogenesis, mRNA stabilisation, regulation of transcription, chromatin decondensation and nucleocytoplasmic transport [39]. What is of additional interest is that it has been shown to have anti-apoptotic activity. This process is accomplished by stabilising *bcl-2 *and *bcl-*_*XL *_at the mRNA level [40-43].

### Protein Biosynthesis

The protein biosynthesis machinery is a complex system of protein-protein, protein-DNA, and protein-RNA interactions. The majority of proteins involved in translational activity were down regulated. For instance, elongation factor 2 was down regulated. This translational elongation factor promotes the GTP-dependent translocation of the nascent protein chain from the A-site to the P-site of the ribosome and ensures high accuracy and speed of the translational mechanism [44]. As an extra note, this protein was also selected for western verification but the rat antibody did not recognize the CHO protein. In addition, both tryptophanyl tRNA synthtase and heterogeneous nuclear ribonucleoprotein (hnRNP A3) are down regulated in cMycCHO. Tryptophanyl tRNA synthtase is involved in catalysing the attachment of an amino acid to the elongation factor 2 cognate transfer RNA molecule and hnRNP A3 implicated in cytoplasmic RNA trafficking [45-47].

Two other proteins involved in protein biosynthesis were up-regulated. Firstly, Translation Factor eIF6, also demonstrated in the western blot (Figure 6), which can regulate translation by modulating the access of the large ribosomal subunits to the elongation cycle, binds to 60S subunits which are activated by release of eIF6 [48]. Overexpression of eIF6 overloads 60S subunits with eIF6 resulting in a dose-dependent inactivation of translational activity and blocking 80S formation [49]. Additionally, eIF6 is required for miRNA silencing of translation [50]. The second up-regulated protein is initiation factor 4A-II (eif4A2), a protein which allows efficient binding of the small ribosomal subunit, and subsequent scanning for the initiator codon and comes from a family that participates in diverse processes apart from translation, including pre-mRNA splicing and ribosome biogenesis [51,52]. Using what has been found about the physiology of CHO cells it could be postulated that this decrease in translational machinery can partly be reflected in the observed decrease in cell size. It already has been put forward that the regulator of cell size, the mTOR pathway, is directly involved in translation control and that the main factors involved in recruiting the ribosome are phosphoproteins whose phosphorylation states are modulated by mTOR pathway proteins [53]. Also, it was shown that the CHO-K1 cell line has a decreased critical mass for initiating entry into S phase of the cell cycle (unpublished data).

### Energy Metabolism

In terms of energy metabolism the proteins ATP synthetase subunit beta and alpha chain which are involved in ATP synthesis have been up-regulated in cMycCHO. These proteins are part of the ATP synthetase complex providing the primary utilizable high energy phosphate bonds in the form of adenosine triphosphate (ATP) for numerous cell processes, acting as an allosteric effector [54]. This could indicate a change in the glycolytic pathway to release more ATP, a possibility supported by similar changes in phosphoglycerate kinase activity which plays a predominant role in the conversion of 3-phosphoglyceroyl phosphate into 3-phosphoglycerate to make ADP into ATP [55]. Pyruvate kinase isozymes M1/M2 (highly active tetrameric form/nearly inactive dimeric form) was up-regulated and this gycolytic enzyme catalyzes the transfer of a phosphoryl group from phosphoenolpyruvate (PEP) to ADP, (subsequently generating ATP) [56] which corresponds with the up-regulation of ATP synthetase subunit beta and alpha chain. For pyruvate kinase isoenzyme M1/M2 the transition between the 2 isozyme forms contributes to the control of glycolysis [57]. Spoden et al, found that M2-PK can act under reduced glucose consumption leading to enhanced cell cycle progression as well as survival, while reducing cell size[58], an observation seen with this cell line. Another up-regulated protein is isocitrate dehydrogenase (IDH), which participates in the tricarboxylic acid cycle, and is found to contribute overall to glutamate synthesis, cellular growth from carbon sources and to mtDNA stability [59,60].

The fact that cMycCHO cell line has decreased lactic acid production is likely the resultant from the down regulated lactate dehydrogenase (LDH) activity. Lactate dehydrogenase catalyses the interconversion of pyruvate and lactate and the suppression of LDH in CHO cells have consequently resulted in increased ATP synthesis [61]. In addition, efficient shuttling of pyruvate into the citric acid cycle which can be responsible for decreased lactate production as well. An interesting note is that specific glucose consumption is slightly lower in cMycCHO. This can be related to the protein Triose-phosphate isomerase being down regulated as a main glycoltyic protein involved in glucose processing in the pathway and increased dimeric M2-PK activity. There was a 50% decrease in lactate production in cMycCHO culture indicating that there could be improved utilisation of glucose to increase ATP output due to aerobic glycolysis being more efficient.

### Cellular Adhesion

Proteins involved in promoting cellular adhesion were all down-regulated in the cMyCHO cell line. One of the down-regulated protein is integrin beta 2 precursor, a protein involved in the integrin mediated pathway with a main functional role in cell to cell adhesion [62]. The integrin beta 2 precursor, although passing the identification criteria set, did not score high, with a cross correlation score of 10.1 but the presence of this protein was supported by the presence of other down regulated proteins involved in adhesion which was seen to be consistent throughout the analysis. Another protein which is down-regulated and known to promote cell adhesion is Galectin-1 (Gal-1). This protein has been shown to increase the adhesion of various cell lines to the extra cellular matrix (ECM) through crosslinking the integrins with ECM components such as laminin and fibronectin [63-65]. Gal-1 also causes the biphasic modulation of cell growth [66,67]. The down-regulated annexin-A2 (ANX2, formerly called annexin II), is a protein encoded by some 20 different genes and promotes enhanced cell to cell adhesion when expressed on the surface of metastatic cells [68]. This protein has multi-functionality since it is shown to be associated with cell-matrix interaction and been suggested to have a role in exocytosis and endocytosis [69]. Early work has briefly looked at the attachment and detachment properties of cMycCHO showing that the cell line was only able to attach 85% of cells onto a substratum surface as a monolayer while the control attached 100% of cells after 200 minutes, while cell detachment rate was shown to be faster than the control [12].

Another protein that has been shown to affect the adhesion of the cell is Calreticulin (CRT). This multifunctional protein is also known to be involved in endoplasmic reticulum (ER) associated pathways but when expressed on the cell surface it can modulate the adhesion properties where it has been reported to complex with integrins [70,71]. Furthermore it is able to bind to laminin [72] and fibrinogen [73], which would explain why CRT down regulation is associated with weaker attachment of the cMycCHO cell line to both of these surface proteins[72,73]. CRT has also been shown to regulate vinculin (a cytoskeletal protein involved in adhesion) in fibroblast cells where its overexpression increased cell to cell adhesion and cell substratum adhesion [74].

Earlier [12] and current work on CHO cell adhesion (data not shown),) together with this proteomic investigation discovering these resultant identified proteins were found to impart the less adhesive phenotype may be further studied to reengineer cell lines with even greater suspension culture adaptation efficiency or to stop the reversion of cell lines to adherent culture as this is the case with many CHO cell lines.

### Cytoskeleton

Several proteins related to cytoskeletal activity were de-regulated in the cMycCHO cell line, such as tropomyosin which is involved in binding actin affecting the assembly and disassembly of actin filaments but its exact mechanism still remains to be determined [75]. Tropomyosin has several isoforms that are cell cycle dependant [76,77]. This relationship with the cell cycle could explain the up-regulation of the protein within the cMycCHO cell line. A cytoskeletal associated protein that recently has been implicated in cell and mitochondrial morphology plus organisation is vimentin. This protein has been suggested to be the intermediate between the mitochondria and microtubules and its depletion results in mitochondrial reorganization and defragmentation [78]. The up-regulation of the dynamically structured vimentin could be related to the up-regulation of proteins involved in ATP and glucose processing discussed earlier, but further studies into the role of this protein and its interaction with the mitochondria will need to be investigated in order to come to a firm conclusion. In previous proteomic studies of CHO cells, vimentin was shown to be up-regulated when the cells where temperature shifted to a lower temperature to induce increased protein secretion rates but decrease cell cycle kinetics [79,80], although the cells seemed to be at a resting state arrested in G0/G1 of the cell cycle, in both cases the proteins related to metabolic energy production pathways were up-regulated and studies involving glucose consumption measurements at lower temperatures confirm these results in CHO cells [81]. This further supports a possible role of vimentin in determining the metabolic state of the cell.

Another cytoskeletal protein, which has been investigated for its role in secretory vesicle transport is F-actin capping protein [82]. F-actin capping protein regulates growth of the actin filament by capping the ends of growing filaments. It is involved in exocytosis of the cell by negatively regulating exocytosis via binding and blocking Syntaxin 4 accessibility [82]. The over-expression of F-actin capping protein has been shown to increase protein secretion [18]. Another proteomic study found that this protein is down regulated 1.5 fold at 37°C after 144 hours but not at 31°C [80], but since these results where in a non-productive cell line the affect this has on productivity could not be concluded. In cMycCHO, F-actin capping protein is also down-regulated giving the possibility that c-Myc overexpression may divert secretory cellular machinery to decrease protein secretion.

### Protein folding

As mentioned before CRT is also involved in the ER processing pathways in addition to its role in cell adhesion processes. CRT is known to function as a molecular Ca^2+ ^binding/storage chaperone involved in the folding of proteins and glycoproteins [83]. It normally resides in the ER lumen where it binds to mis-folded proteins and prevents them from being exported from the ER to the Golgi apparatus. It has been shown, using fluorescently labeled proteins, that calreticulin interacts with protein disulfide isomerase (PDI) in a Ca^2+^-dependent manner [84]. As it happens PDI is also down regulated in cMycCHO cells along with another ER protein; endoplasmin precursor (GRP94). The other identified ER protein was GRP78, which was found to be up-regulated, both in the 2D analysis (Figures 4 and 5) and western detection assay (Figure 6). The ER lumenal molecular chaperones known to be involved in monoclonal antibody synthesis and assembly are PDI, GRP94, and GRP78 [85]. The effect this has on protein secretion has been shown to be cell type and protein specific [86-89]. This might indicate that cMyc has an effect on the productivity, but it must not be ruled out that it may be dependent on the type of protein secreted as well, and thus further investigation would be needed to address this particular question. Thioredoxin was found to be down regulated, and since this protein is known to reduce disulfide bonds in target protein it may affect protein production/secretion rates. Thioredoxin is also involved in DNA synthesis as a hydrogen donor for ribonucleotide reductase [90]. Since cell division has increased for the cMycCHO cell line, it is likely that a reduction in protein folding could, partly, be due to down-regulated thioredoxin in the cytoplasm, meaning less thioredoxin is available for this particular, process rather then the thioredoxin associated with DNA synthesis. A future study using cellular fractionation might give further insight into the function of this protein in the folding pathway.

Another protein involved in protein folding is the 60 kDA heat shock protein (HSP60) which was found to be up-regulated. This is a mitochondrial chaperone responsible for the transportation and refolding of proteins from the cytoplasm into the mitochondrial matrix [91]. Since mitochondrial associated proteins that have been identified related to increases in energy production are up-regulated it might explain the increase in HSP60 to help the mitochondrial function optimally. HSP60 may also play a key role in anti-apoptotic activity by binding to BAX and Bak [92]. Another anti-apoptotic contributor with multi-functionality identified in the proteome profile is PRDX2. This is an antioxidant enzyme that uses its cysteine residues to breakdown reactive oxygen species (ROS) and found to have chaperone activity as well, when the cell is under stressful conditions [93]. A reduced PRDX2 has limited capacity to eliminate ROS but a chaperone activity becomes more prominent at this stage [94]. To survive at higher cell densities than the control, cMycCHO must be able to augment its internal machinery to prevent apoptosis occurring while growth takes place beyond the control cell line. The increase in PRDX2 and HSP60 activity both relate to cell survival and HSP60, additionally supports the mitochondrial proteins to work efficiently by being a mitochondrial chaperone.

A protein also known to affect apoptotic pathways is Cytochrome C, which was down-regulated in cMycCHO culture. This protein is known for sensitizing the cell towards the mitochondrial activated pathway for apoptosis and could help explain why the cMycCHO cell appeared to have a longer stationary phase culture (with viability above 90%).

## Conclusion

The present study has identified several proteins in an enhanced-proliferation cell line with increased maximal cell density that can relate to the phenotype that may be used as future targets in CHO cell engineering strategies. Changes in proteins involved in energy metabolism appear to be related to the increased cell density. Also, the decreased lactate production can be explained by the down regulation of LDH. Moreover, this study gives further insights into the mechanism related to the down regulation of adhesion proteins by c-Myc that may form the bases for developing an anchorage independent CHO cell lines.

## Competing interests

The authors declare that they have no competing interests.

## Authors' contributions

DK, MJD and MAR designed the research. DK performed all the experiments and analyzed the data. MAR conceived the study. DK and MAR wrote the manuscript. All authors read and approved the final manuscript.

## Supplementary Material

Additional file 1 **Table S1 displaying protein species with a changed regulation for cMycCHO**. A list of protein species with a changed regulation level for cMycCHO versus the CHO-K1 control cell line. XCorr Score is an abbreviation for the raw cross-correlation score of the top candidate peptide or protein for a given input data file. The higher the XCorr Score, the better the match to the searched sequence at a probability cut off (P > 0.001). Note that Xcorr values in combination with the search parameters rather than the matched peptides should be considered as significant hits. Click here for file
