# Supplementary material for: A proteomic study of cMyc improvement of CHO culture
Source: BMC Biotechnol. 2010 Mar 22;10:25. doi: 10.1186/1472-6750-10-25 (PMC2859402; doi:10.1186/1472-6750-10-25)
Supplement: Additional file 1 — Table S1 displaying protein species with a changed regulation for cMycCHO. A list of protein species with a changed regulation level for cMycCHO versus the CHO-K1 control cell line. XCorr Score is an abbreviation for the raw cross-correlation score of the top candidate peptide or protein for a given input data file. The higher the XCorr Score, the better the match to the searched sequence at a probability cut off (P > 0.001). Note that Xcorr values in combination with the search parameters rather than the matched peptides should be considered as significant hits. [file 1472-6750-10-25-S1.DOC]

**Table 1**
